# Synergistic action of inflammation and lipid dysmetabolism on kidney damage in rats

**DOI:** 10.1080/0886022X.2018.1450763

**Published:** 2018-03-23

**Authors:** Zhongjie Wang, Wenhan Huang, Hui Li, Lin Tang, Hang Sun, Qi Liu, Ling Zhang

**Affiliations:** a Chongqing Medical University, Chongqing, China;; b Department of Rheumatology and Immunology, The Second Affiliated Hospital of Chongqing Medical University, Chongqing, China;; c Yan’an Hospital Affiliated to Kunming Medical University, Yunnan, China;; d Key Laboratory of Molecular Biology for Infectious Diseases, Ministry of Education, The Second Affiliated Hospital of Chongqing Medical University, Chongqing, China;; e Department of Nephrology, The Second Affiliated Hospital and Center of Lipid Research of Chongqing Medical University, Chongqing, China

**Keywords:** Adriamycin, lipid, nephrosis, transforming growth factor-β1 (TGF-β1), tumor necrosis factor-α (TNF-α)

## Abstract

In kidney disease, inflammation and lipid dysmetabolism are often associated together, however, the effect and mechanism of inflammatory mediators and lipid dysmetabolism on kidney damage is still unclear. In this study, Wistar rats were randomized into four groups: normal diet + saline (Group N), high-fat diet (HF)+ saline (Group HF), normal diet + adriamycin (Group ADR), HF + adriamycin (Group ADR + HF). After 10 weeks of feeding, rats in each group were randomly sacrificed. We found that the protein content of urine in ADR and ADR + HF groups were significantly higher than that of group N and HF while the serum levels of total protein and albumin in the ADR and ADR + HF groups decreased correspondingly. The serum levels of triglyceride, total cholesterol and low-density lipoprotein in the HF, ADR and ADR + HF groups increased. In the treatment groups, mesangial proliferation, matrix accumulation, tubular vacuolization, inflammatory cell infiltration and fat deposition were detected. These pathological changes were the most serious in the ADR + HF group. The expression of tumor necrosis factor-α (TNF-α) and transforming growth factor-β1 (TGF-β1) were increased in each treatment group, especially in the ADR + HF group. Our results suggested that the inflammatory factors and abnormal lipid levels can activate the inflammatory response in kidney of the Wistar rats, and lead to a series of pathological changes in renal tissue, and inflammatory factors and lipid dysmetabolism can aggravate damage in the kidney.

## Introduction

Glomerulosclerosis and renal interstitial fibrosis is the common pathway for chronic kidney disease and kidney failure [1]. Since 1982, when Moorhead et al. [2] put forward the hypothesis of ‘lipid nephrotoxicity’ for the first time, a large number of studies have confirmed that chronic kidney diseases are often accompanied by various lipid abnormalities. While lipid dysmetabolism can also cause the progression of kidney disease and glomerulosclerosis [3,4], the mechanism of lipid-induced renal damage has not yet been clarified.

Recent experimental and clinical evidence has confirmed that the pathological changes and pathophysiological mechanism of glomerulosclerosis are similar to those of atherosclerosis, and it has put forward the concept ‘glomerular atherosclerosis’ [5,6]. Recent studies have confirmed that atherosclerosis is an inflammatory disease and that inflammation is an accelerating factor for it; at the same time, the levels of inflammatory factors are found to be higher in many kinds of kidney diseases [7]. Adriamycin nephrosis is a representative animal model of nephrotic syndrome, pathologically characterized by extensive tubular injury, interstitial inflammation and renal fibrosis [8].

TNF-α is a cytotoxic factor, it plays a key role in the pathogenesis of fibrosis, it is involved in many inflammatory responses, which can induce the release of many cytokines and functions as a chemotactic molecule to recruit neutrophils and monocytes [9]. TGF-β1 is the well-known fibrogenic factors, TGF-β1 combining with its receptor leads to the activation of its downstream component Smad3. Massive researches confirmed that TGF-β1/Smad3 signaling pathway was activated in a high lipid circumstance [10,11]. Through the TGF-β1/Smad3 signaling pathway, it would result in cell proliferation and fibrosis, stimulates the mesangial cell collagen synthesis [12,13].

In this research, we attempted to study the role of inflammatory factors and lipid dysmetabolism in the promotion of glomerulosclerosis in Wistar rats.

## Methods

### Establishment of models and experimental periods

The experiments were approved by the local ethics committee. Forty-eight male Wistar rats (10 ∼ 11 weeks old, 240 ∼ 275 grams, from Center for Experimental Animals of Chongqing Medical University) were kept in animal’s rooms where the temperature was between 22 °C and 28 °C and the light/dark cycle (L/D) was 12 h (6 AM to 6 PM). Before the experiments, the rats were fed a normal diet.

The 48 rats were randomly assigned to four groups (12 rats in each group): (a) The normal control group (Group N) rats were fed the normal diet and injected with 0.9% normal saline (N.S 3 mL/kg) in the tail vein. (b) The high-fat diet group (Group HF) rats were fed a high-fat diet (containing 60% kcal fat, 4% cholesterol from the Guangzhou Tianma Fine Chemical Plant and 1% sodium cholate from Beijing Aoboxing (Universeen Bio-tech Co. Ltd, Beijing, China) and injected with 0.9% N.S (3 mL/kg) in the tail vein. (c) The adriamycin nephrosis group (Group ADR) rats were fed the normal diet and injected with 2 mg/ml doxorubicin hydrochloride (6 mg/kg, from the Pharmacia Corporation) in the tail vein [14]. (d) The adriamycin nephrosis plus high-fat diet group (Group ADR + HF) rats were fed the high-fat diet and injected with 2 mg/ml doxorubicin (6 mg/kg) in the tail vein. Rats in each group were randomly sacrificed at week 12.

### Urine and serum biochemical tests

At week 0 and 12, proteinuria was measured using the Bradford method and then 24 h urine protein excretion (24hUPE) was calculated. Serum was collected at week 12. Serum total protein (TP), albumin(ALB), blood urine nitrogen (BUN), serum creatinine (Scr), triglyceride (TG), total cholesterol (TC), low-density lipoprotein (LDL) were determined by an automatic analyzer (Model 7020; Hitachi, Tokyo, Japan)

### Histological procedures

The kidneys were immersed in 10% neutral buffered formalin, and then were embedded in paraffin. Sections were then stained with hematoxylin-eosin (HE), periodic acid-Schiff (PAS) and Sudan III stain. Detection of TNF-α was performed by an immunohistochemical method according to Cell Signaling Technology’s Protocol. Rabbit polyclonal anti-TNF-α was used as the primary antibody. Horseradish peroxidase-linked sheep anti-mouse IgG was used as the second antibody.

For electron microscopy, small blocks of kidney were fixed in 2.5% buffered glutaraldehyde and fixed in 2% osmium tetroxide, dehydrated with graded ethanol and then embedded in epoxy resin. The ultrastructure of kidneys was then observed with an electron microscope.

### Analysis of serum tumor necrosis factor-α (TNF-α) by ELISA and detection of the protein expression of TNF-α and TGF-β by the Western blotting method

At week 12, Serum TNF-α was detected by an enzyme-linked immunosorbent assay kit (ELISA kit, JingMei Biological Engineering Co. Ltd, China). Monoclonal antibodies to rat TNF-α were added to a 96-well plate firstly. Then test samples and the standard solutions were added to the plate. After TNF-α of biotinylated monoclonal antibodies to rat TNF-α were added, streptavidin-alkaline phosphatase was added for the reaction. Diaminobenzidine was added to induce the color reaction. An automated microplate reader set at 450 nm was used to measure the optical density. A standard curve was drawn by plotting optical density versus the log of the concentration of standard solution.

The protein extracted from the homogenates of the kidneys was used to analyze the TNF-α and TGF-β expression by western blot. The concentration of protein was measured by the Bradford method and adjusted to the same volume of sample buffer. The samples were electrophoresed (25 µg/lane) in an acrylamide denaturing sodium dodecyl sulfate (SDS)–polyacrylamide gel, transferred to a PVDF membrane and the membrane was incubated with 1 µg/mL TNF-α or TGF-β rabbit polyclonal antibody (Wuhan Boster Biological Technology Co. Ltd, Wuhan, China). The membrane was then incubated with horseradish peroxidase-linked sheep anti-rabbit IgG (Zhongshan Goldenbridge Biotechnology Co. Ltd, Guangzhou, China) at a 1:1000 dilution. After three times washed by Tris-buffered saline-Tween (TBST), the proteins were measured by enhanced chemiluminescence (ECL) kit (KeyGEN Biotech, China) on Bio-Rad ChemiDoc Imaging System. All densitometric results were reported normalized to β-actin.

### The mRNA expression of TNF-α and TGF-β in renal tissue was detected by real-time PCR method

The total RNA was extracted from the kidney tissue using an RNA isolating reagent kit (Shanghai Fastagen Biological Engineering Co. Ltd, Shanghai, China) and then used as a template for reverse transcription using the RT reagent Kit (TaKaRa, Japan). The real-time-PCR was performed with the ABI 7300 real-time PCR System (Applied Biosystems), using β-actin as a control. The following primers were used:

**TGF-β**

Foward primer 5-CAATGGGATCAGTCCCAAAC-3

Reverse primer 5-TTCTCTGTGGAGCTGAAGCA-3

**TNF-α**

Foward primer 5-AGTTGGGGAGGGAGACCTT-3

Reverse primer 5-CATCCACCCAAGGATGTTTAG-3

**β-actin**

Foward primer 5-CTCTTCCAGCCTTCCTTCCT-3

Reverse primer 5-TAGAGCCACCAATCCACACA-3

Following the supplier’s manual (the real-time-PCR reagent kit was from the TAKARA Company), the real-time-PCR reactions were carried out in automated thermocyclers by incubation at 95 °C for 30 s, followed by 40 cycles of 5 s at 95 °C and 31 s at 60 °C as stage two for PCR. Relative gene expression was calculated by using the 2–△△CT Method and the relative mRNA expression was estimated by normalization with β-actin.

### Statistical analysis

All of the measurement data are presented as mean ± standard deviation (SD). The data were statistically analyzed by SPSS12.0 (SPSS Inc., Chicago, IL). Differences within each group were tested by one-way analysis of variance (ANOVA) and the Student–Newman–Keuls test. *p* < .05 was considered statistically significant.

## Results

### Biochemical indicators in rats with hyperlipidemia and inflammation

**Figure 1.**
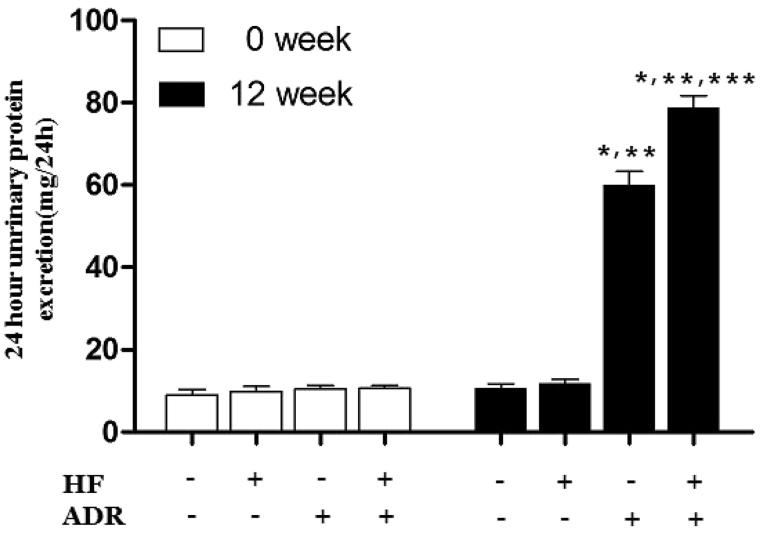
24-h urinary protein excretion for rats in each group. Data are shown as mean ± SD. Compared with Group N: *p < .05; compared with Group HF: **p < .05; compared with Group ADR: ***p < .05. *n* = 4.

In this study, we utilized a group of biomarkers to assess the lipid and protein metabolism as well as renal function, including the 24hUPE, serum levels of TG, CHOL, LDL, TP, ALB, BUN and Cr, as shown in Figure 1 and Table 1. It was demonstrated that no significant differences in BUN or Scr were seen between groups (*p* > .05). In Group HF, high-fat diet induced hyperlipidemia, serum level of TG, TC and LDL were increased comparing with that in Group N. (TG: Group HF: 3.52 ± 0.60 mmol/L versus Group N: 1.37 ± 0.37 mmol/L, *p < .05; TC: Group HF: 5.33 ± 0.45 mmol/L versus Group N: 1.93 ± 0.16 mmol/L, *p < .05; LDL: Group HF 3.03 ± 0.59 mmol/L versus Group N 0.60 ± 0.17 mmol/L, *p < .05). In group ADR, Adriamycin induced proteinuria, hypoalbuminemia (24hUPE: Group ADR: 59.93 ± 3.36 mg/24 h versus Group N: 10.71 ± 1.00 mg/24 h, *p < .05; TP: Group ADR: 42.39 ± 3.72 g/L versus Group N: 58.87 ± 7.77 g/L, *p < .05; ALB: Group ADR: 22.37 ± 1.44 g/L versus Group N: 39.16 ± 2.39 g/L, *p < .05) and hyperlipidemia (TG: 4.64 ± 0.73 mmol/L versus Group N, *p < .05; TC: 6.38 ± 0.65 mmol/L versus Group N, *p < .05; LDL: 2.21 ± 0.70 mmol/L versus Group N, *p < .05).

**Table 1.** Concentration of serum BUN, cr, TP, ALB, TC, TG and LDL for rats in each group.

Group	BUN (mmol/L)	Scr (μmol/L)	TP (g/L)	ALB (g/L)	TG (mmol/L)	TC (mmol/L)	LDL (mmol/L)
N	6.42 ± 0.77	113.36 ± 3.56	58.87 ± 7.77	39.16 ± 2.39	1.37 ± 0.37	1.93 ± 0.16	0.60 ± 0.17
HF	6.32 ± 0.53	114.28 ± 2.22	63.02 ± 9.67	38.21 ± 1.56	3.52 ± 0.60*	5.33 ± 0.45*	3.03 ± 0.59*
ADR	6.72 ± 0.90	116.72 ± 6.73	42.39 ± 3.72*	22.37 ± 1.44*	4.64 ± 0.73*	6.38 ± 0.65*	2.21 ± 0.70*
ADR + HF	6.86 ± 0.96	117.86 ± 4.20	34.43 ± 2.49*^,^**^,^***	19.84 ± 2.05*^,^**^,^***	5.92 ± 0.87*^,^**^,^***	10.59 ± 1.03*^,^**^,^***	5.71 ± 0.60*^,^**^,^***

Data are shown as mean ± SD.

* *p* < .05 versus Group N.

** *p* < .05 versus Group HF.

*** *p* < .05 versus Group ADR.

### Pathological changes in renal tissue

**Figure 2.**
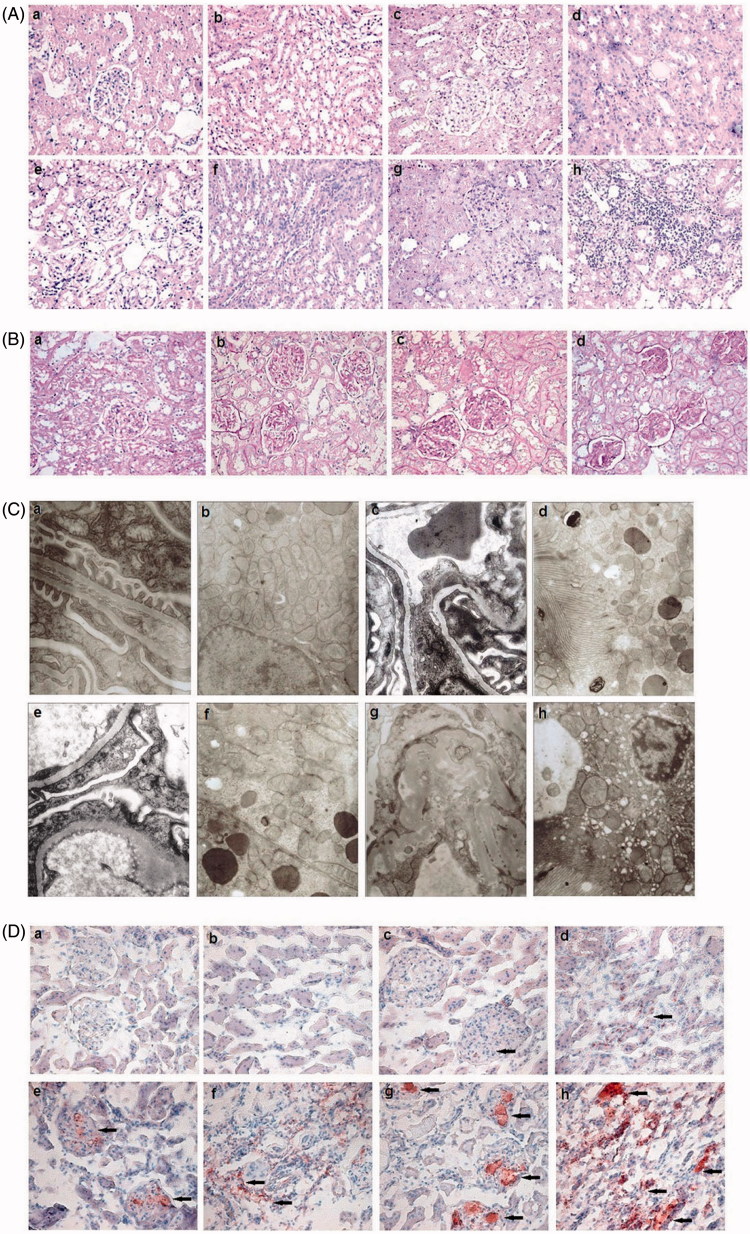
Pathological changes in renal tissue. (A) HE staining of the glomeruli and tubules in Group N (a and b), HF (c and d), ADR (e and f), ADR + HF (g and h), (400×). (B) PAS staining of the glomeruli in Group N (a), HF (b), ADR (c), ADR + HF (d), (400×). (C) The ultrastructure of the glomeruli and tubules detected by electron microscopy, in Group N (a and b), HF (c and d), ADR (e and f), ADR + HF (g and h) (Glomeruli EM 14,000×, Renal tubules EM 7000×). (D) Sudan III staining of glomeruli and tubules in Group N (a and b), HF (c and d), ADR (e and f), ADR + HF (g and h), (400×). Immunocytochemistry showed no fat expression in the kidneys of Group N, little fat expression in the tubular epithelium of Group HF, more fat expression in the epithelium of glomeruli and renal tubules in Group ADR, Strong fat expression in glomeruli and renal tubules in Group ADR + HF (arrowheads).

We evaluated pathological changes under light microscope and electron microscope. As shown in Figure 2(A–D), in Group N, it did not reveal any changes by light and electron microscopy. In group HF, high-fat diet induced slightly increased glomerular size, wide mesangial matrix, incrassated segmental and focal basement membrane, fused foot processes. The pathological changes, which including glomerular expansion, endocapillary hypercellularity, mesangial matrix expansion, mesangial cells proliferation, glomerular basement membrane thickening, foot processes fusion, tubular epithelium loosening, inflammatory cell infiltration in Group ADR were significantly more serious than those in Group HF. In Group ADR + HF, foam cells were observed in mesangial areas, and some glomeruli had progressed to form focal and segmental glomerulosclerosis. The extent of the pathological changes in Group ADR + HF were the most serious among the groups. By staining with Sudan III, we found there was no lipid deposited in the kidneys in Group N. Fat deposited in the kidneys of the rats in the treatment groups. In addition, the fat deposition in Group ADR + HF was higher than that of Group HF and Group ADR.

### TNF-α level in serum detected by ELISA. TNF-α level in renal tissue detected by immunohistochemistry, Western blotting and real-time PCR method

**Figure 3.**
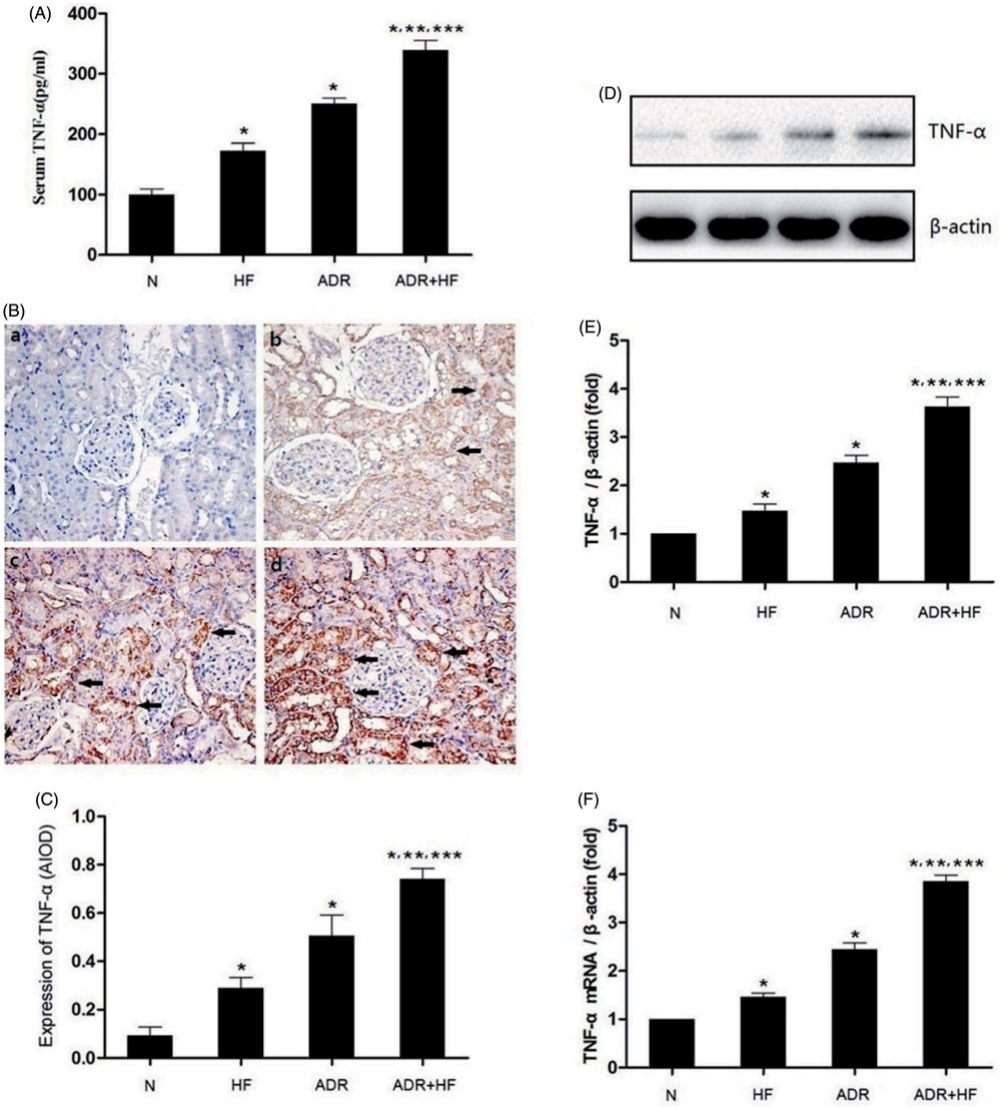
TNF-α level in serum detected by ELISA. TNF-α level in renal tissue detected by immunohistochemistry, western blotting and Real-time PCR method. (A) Level of serum TNF-α in each group. Compared with Group N: *p < .05; compared with Group HF: **p < .05; Compared with Group ADR: ***p < .05. *n* = 5. (B, C, D, E and F) The expression of TNF-α in the renal tissue of each group was detected by immunohistochemistry (400×) (arrowheads) (B and C), western blotting (D and E) and Real-time PCR (F). There is scant TNF-α expression in Group N and more in the other three groups. The expression of TNF-α in Group ADR was more than that in Group HF. Among the groups, ADR + HF had the highest expression of TNF-α. Compared with Group N: *p < .05; compared with Group HF: **p < .05; Compared with Group ADR: ***p < .05. *n* = 5–6.

Our results showed that the expression of the TNF-α in Group HF (172.48 ± 12.49 pg/ml), ADR (250.19 ± 9.63 pg/ml) and ADR + HF (339.19 ± 16.06 pg/ml) was higher than that in Group N (ADR + HF > ADR > HF ≫ N) (99.68 ± 9.58 pg/ml) both in serum (Figure 3(A)), and the same results were obtained in the renal tissue by the immunohistochemistry technique (Figure 3(B,C)).

Western blot suggested that the level of expression of TNF-α in Group HF and Group ADR were higher than that of Group N (TNF-α: Group HF: 1.47 ± 0.14-fold of Group N, *p < .05; Group ADR: 2.47 ± 0.15-fold of Group N, *p < .05). However, TNF-α in Group ADR + HF was significantly higher than that of Group HF and ADR. (*p < .05) (Figure 3(D,E)). Besides, the expression of TNF-α mRNA was consistent with the TNF-α protein expression (Figure 3(F)).

### Expression of TGF-β1 were detected by real-time-PCR and Western blotting in renal tissue

**Figure 4.**
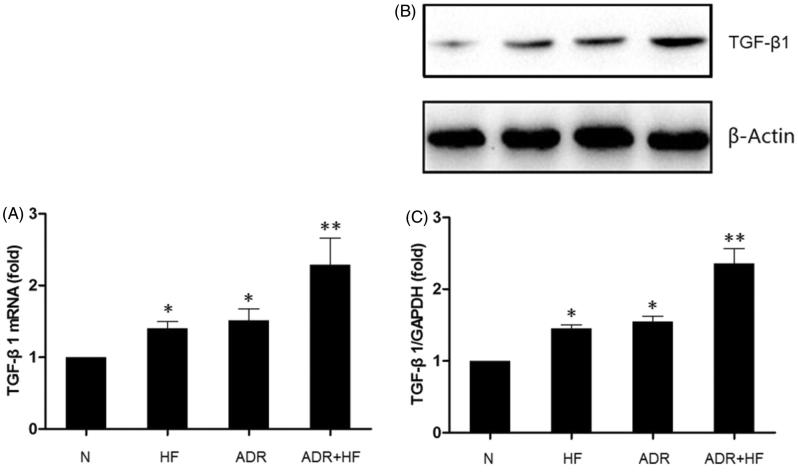
Expression of TGF-β1 in renal tissue. (A, B and C) The expression of TGF-β1 in the renal tissue of each group was detected by Real-time PCR (A), and western blotting (B and C). The expression of TGF-β1 in Group HF and Group ADR were more than that in Group HF. Among the groups, ADR + HF had the highest expression of TGF-β1. Compared with Group N: *p < .05; compared with Group HF and Group ADR: **p < .05. *n* = 5–6.

The results of real-time PCR and western blot (Figure 4(A–C)) revealed that high-fat diet induced the up-regulation of the TGF-β1 expression (real-time PCR: Group HF: 1.36 ± 0.13-fold of Group N, *p < .05; western blotting: Group HF: 1.47 ± 0.09-fold of Group N, *p < .05). Compared with the Group N, adriamycin also significantly increased TGF-β1 expression (real-time PCR: Group ADR: 1.51 ± 0.16-fold of Group N, *p < .05; western blotting: Group HF: 1.62 ± 0.13-fold of Group N, *p < .05). The TGF-β1 expression in Group ADR + HF was higher than that in both Group HF and Group ADR.

## Discussion

Moorhead’s research team revealed that hyperlipidemia may be potentially nephrotoxic [2]. Subsequently, increasing evidence has showed that lipids is a risk factor for renal injury [15–17]. Although ‘lipid nephrotoxicity’ has been widely accepted and confirmed by some animal experiments [18], the level of plasma cholesterol is not consistent with the extent of glomerular sclerosis, which suggests that some factors other than lipids are also related to glomerulosclerosis. Recent research has been revealed that other forms of glomerular injury that promoted the development of glomerulosclerosis, including preexisting glomerular disease, or the presence of hypertension [19,20]. Furthermore, some evidence has suggested that inflammation aggravated the lipid-mediated peripheral cell injury, which has many similarities to atherosclerosis [2,5].

Our results showed high-fat diet induced hyperlipidemia and slight renal injury. A single dose of ADR induced the rat’s nephrotic syndrome (NS), which was characterized by heavy proteinuria, hypoalbuminemia, anasarca and hyperlipidemia. Histological changes revealed endocapillary hypercellularity, mesangial matrix expansion, mesangial cells proliferation, glomerular basement membrane thickening, focal loss of the normal arrangement and architecture of the foot processes, tubular epithelium loosening, inflammatory cell infiltration. The morphological changes of the kidneys in rats administered ADR were consistent with previously described changes [21]. These results suggest that we successfully established the lipid-induced renal damage model and ADR-induced nephrotic syndrome model with Wistar rats. Moreover, we demonstrated that the proteinuria, hypoalbuminemia, and hyperlipidemia of the nephrotic syndrome and pathological changes in Group ADR + HF were more severe than those in Group ADR. Our findings indicate that the biochemical indicators and pathological changes in rats with hyperlipidemia and inflammation are more serious than those in rats with hyperlipidemia or inflammation separately, which suggests that the synergy between hyperlipidemia and inflammation can cause more serious renal damage.

It has been well demonstrated that TGF-β1 is a key factor in the development of inflammation and leading to fibrosis in many kinds of kidney diseases [22,23]. TNF-α, the inflammatory mediator, has been proved to promote the glomerular hypertrophy, expansion of the mesangial matrix and thickening of the glomerular and tubular basement membranes, ultimately resulting in proteinuria, glomerulosclerosis and tubulointerstitial fibrosis [24]. Zhao’s research team demonstrated that the Wistar rats with high-fat diet induced a significant increase in TNF-α and TGF-β expression both in mRNA level detected by real-time PCR and in renal tissue detected by immunohistochemistry [25].

Our research also proved that fed Wistar rats with high-fat diet up-regulated the expression of TNF-α and TGF-β1, suggesting the inflammatory factor TNF-α and TGF-β1 were activated in a hyperlipidemia circumstance. Moreover, this study revealed that expression of the TNF-α and TGF-β1 were also increased after the rats were suffered from inflammation, the results were consistent with other researches [26,27]. The inflammatory cytokines, such as TNF-α and TGF-β1, were involved in kidney damage [28,29]. Finally but importantly, we first found that Wistar rats administered ADR and fed with high-fat diet sharply increased the TNF-α and TGF-β1 expression, and the extent of the pathological changes in Group ADR + HF were the most serious. This indicated that in ADR induced the rat’s nephrotic syndrome, dyslipidemia leaded to the further damage of the kidney, and those changes were mediated through the elevated TNF-α and TGF-β1.

In conclusion, our study proved that the rat’s nephrotic syndrome could be induced by ADR, which was characterized by heavy proteinuria, hypoalbuminemia. In addition, increased lipid levels may activate the renal inflammatory response. Finally, there are synergies between lipid dysmetabolism and inflammation in the kidney, and this synergies between inflammation and lipid dysmetabolism cause more serious renal damage than either inflammation or lipid dysmetabolism does separately.
